# The long and sinuous road to phage-based therapy of *Clostridioides difficile* infections

**DOI:** 10.3389/fmed.2023.1259427

**Published:** 2023-08-23

**Authors:** Andrew A. Umansky, Louis Charles Fortier

**Affiliations:** Department of Microbiology and Infectious Diseases, Faculty of Medicine and Health Sciences, Université de Sherbrooke, Sherbrooke, QC, Canada

**Keywords:** *Clostridioides (Clostridium) difficile* infection, bacteriophage (phage), phage therapy, endolysin, diffocin, avidocin, phage engineering

## Abstract

With the antibiotic crisis and the rise in antimicrobial resistance worldwide, new therapeutic alternatives are urgently needed. Phage therapy represents one of the most promising alternatives but for some pathogens, such as *Clostridioides difficile*, important challenges are being faced. The perspective of phage therapy to treat *C. difficile* infections is complicated by the fact that no strictly lytic phages have been identified so far, and current temperate phages generally have a narrow host range. *C. difficile* also harbors multiple antiphage mechanisms, and the bacterial genome is often a host of one or multiple prophages that can interfere with lytic phage infection. Nevertheless, due to recent advances in phage host receptor recognition and improvements in genetic tools to manipulate phage genomes, it is now conceivable to genetically engineer *C. difficile* phages to make them suitable for phage therapy. Other phage-based alternatives such as phage endolysins and phage tail-like bacteriocins (avidocins) are also being investigated but these approaches also have their own limitations and challenges. Last but not least, *C. difficile* produces spores that are resistant to phage attacks and all current antibiotics, and this complicates therapeutic interventions. This mini-review gives a brief historical overview of phage work that has been carried out in *C. difficile*, presents recent advances in the field, and addresses the most important challenges that are being faced, with potential solutions.

## Introduction

*Clostridioides difficile* is one of the top priority pathogens according to the US CDC (1). This Gram-positive, strictly anaerobic spore-forming bacillus is the main cause of antibiotic-associated diarrhea. In the early 2000s, major outbreaks occurred in North America and Europe (2). One group of strains, designated as ribotype 027 (R027), has been associated with increased disease severity, poorer clinical outcome, and more frequent relapses (3–5). Current treatments rely on antibiotics, which further disrupt the protective gut microbiota. Spores are resistant to all antibiotics, and once antibiotherapy is stopped, residual spores within the gut or spores ingested from contaminated environments can germinate due to the permissive microbiota. Consequently, many patients experience one or more relapses leading to recurrent *C. difficile* infections (rCDI) (6). The best intervention to treat rCDI is the fecal microbiota transplant (FMT) that swiftly restores the gut microbiota diversity, which is associated with colonization resistance (7). However, this approach presents several limitations including the risk for potential transfer of unwanted microbes and the lack of knowledge on the long-term impact of FMT on health (8). Therefore, other therapeutic strategies are urgently needed. Phage therapy is the administration of bacteriophages (or phages) that specifically kill target bacteria and is a promising alternative or complement to antibiotherapy in the fight against multidrug-resistant pathogens (9–12). The main advantage of therapeutic phages is their great specificity toward target bacteria, thus sparing other beneficial bacteria. A targeted approach like phage therapy could be a very powerful solution in the case of rCDI. Over the last two decades, the potential of phages or phage derivatives to treat or prevent CDI has been explored. This mini-review summarizes the current advances in phage-based approaches to fight against *C. difficile*. The most urgent challenges that must be addressed and mitigating strategies are also discussed.

## Whole phage treatment

Several phages infecting *C. difficile* have been isolated, but only 33 of them have been sequenced and characterized more deeply. It is important to stress that all *C. difficile* phages described to date have a temperate lifestyle, i.e., these phages lead to lysogeny (13, 14). Upon incorporation of a prophage, a bacterial host becomes automatically resistant to further lytic reinfection by the same or a related phage. It is, therefore, generally discouraged to use temperate phages for therapy, although genetic engineering has the potential to transform temperate phages into an important source of therapeutic agents (15, 16). Moreover, phage resistance due to lysogeny can be mitigated or even eliminated by the temperate phage antibiotic synergy (tPAS) phenomenon, which consists of combining temperate phages with sub-inhibitory concentrations of antibiotics to activate the SOS response and prevent phage entry into the lysogenic cycle (17). tPAS has not been tested in *C. difficile*, but the efficacy of several unmodified temperate phages was assessed under different laboratory settings and in preclinical animal models. Table 1 summarizes these studies and those in which phage-derived antimicrobials were tested.

**Table 1:** Relevant studies investigating the use of phage-based therapeutics against *C. difficile*.

**Treatment/model**	**General outcome, limitations/challenges**	**Reference**
**Whole phages**		
CD140; Hamster Model	Phage treatment led to greater hamster survival. Phage resistance arose in one animal, phage treatment did not protect from subsequent *C. difficile* challenge.	(18)
phiCD27; *in vitro* batch fermentation assays	Significant reduction in viable *C. difficile* counts, especially in a prophylactic regimen, higher MOI improved treatment, phiCD27 was specific to *C. difficile* strain. Lysogeny was a probable cause of *C. difficile* growth rebound (5/5 *C. difficile* clones isolated at an MOI of 7 were lysogens).	(19)
phiCD27; Human *in vitro* gut model	Prophylactic treatment with phage cleared *C. difficile* vegetative cells and decreased toxin production, but spore production was higher. Lysogens were isolated from a replicate where phage treatment failed, and higher spore formation was observed during phage treatment.	(20)
phiCDHM1, phiCDHM2, phiCDHM5, phiCDHM6; *in vitro* assays; Hamster model	*In vitro assays:* Dual-phage cocktails reduced lysogeny while three- or four-phage cocktails better prevented lysogeny. *In vivo assays:* Treatment increased infected hamster longevity and reduced *C. difficile* colonization and spore formation but did not protect them from death. Cocktails did not lead to phage resistance. Single- or dual-phage therapy could lead to lysogeny and phage resistance.	(21)
phiCDHM1, phiCDHM2, phiCDHM5, phiCDHM6; *in vitro assays; G. mellonella* model	*In vitro assays:* Phages penetrated biofilm *in vitro* and killed biofilm resident bacteria. *In vivo assays:* Prophylactic application resulted in the survival of larvae, phage treatment reduced *C. difficile* colonization. Phage remedial treatment did not prevent the death of larvae.	(22)
phiCDHM1, phiCDHM2, phiCDHM5, phiCDHM6 phage cocktails; *in vitro* batch fermentation model	*In vitro assays:* Administration of the cocktail-cleared *C. difficile* from culture during remedial and prophylactic regimen, no *C. difficile* regrowth. Phage treatment did not affect other bacterial species.	(23)
phiCDHS1; *in vitro* colonic epithelial cell model	Phage treatment was more effective in the presence of HT-29 cells, reduced *C. difficile* adherence, and phage adsorption was observed on HT-29 cells. Did not consider the mucus layer normally present in the colon.	(24)
Wild-type phiCD24-2, engineered phiCD24-2 (carrying CRISPR-Cas3 components); *in vitro assays*; Mouse model with single phage therapy	*In vitro assays:* Reduced or no lysogeny was observed for modified phages, crPhage killed higher counts of vegetative cells and delayed culture rebound of culture. *In vivo assays*: Engineered phage showed a higher reduction in vegetative cells in feces and intestinal bacterial load than WT phage, reduction of lysogeny in engineered phages. Lysogeny still occurred in a phage deleted of key lysogeny genes and CRISPR components.	(25)
Cocktail of phiCDHM1, phiCDHM2, phiCDHM5 and phiCDHM6; *Galleria mellonella* model	Prophylactic phage application improved larvae survival, reduced bacterial colonization, lowered toxin levels, and remedial regimen delayed larvae death. Phage remedial treatment only delayed larvae death.	(26)
**Diffocins/avidocins**		
Diffocin 4 and diffocin 16; *in vitro assays*	Could be recombinantly expressed in *B. subtilis*. Narrow host spectrum.	(27)
Av-CD291.2 (modified R-type bacteriocin); *in vitro assays;* Mouse model of CDI	*In vitro assays:* Av-CD291.2 had a broader activity on multiple ribotype 027 strains than WT diffocins 4. *In vivo:* Prophylactic administration of Av-CD291.2 inhibited *C. difficile* colonization, and Av-CD291.2 did not affect the mouse gut microbiota. Large-scale production and stability in the gut environment will be challenging.	(28)
Diffocins derived from RT078 *C. difficile* isolate (HMC114) and Av-CD291	*In vitro:* HMC114 can kill 21/25 ribotype 027 isolates tested while Av-CD291 killed all ribotype 027 isolates tested. Diffocins could kill strains that produced them.	(29)
**Phage endolysins**		
phiCD27 endolysin (CD27L); *in vitro* assays	PhiCD27 endolysin can be used to lyse *C. difficile* cells in culture. Endolysin was specific to *C. difficile* and had a broader host range than its parent phage. Endolysin activity was weaker compared with endolysins produced by other phages that infect other species.	(30)
Endolysin catalytic domain (CD27L1-179); *in vitro* assays	Removal of the CBD domain increased the activity of endolysin Removal of the CBD slightly decreased specificity, which caused the lysis of other bacterial species.	(31)
Recombinantly expressed catalytic domain of endolysin PlyCD (PlyCD1-174); *in vitro* assays; *ex vivo* assays	*In vitro assays:* Greater (>4-logs) activity and broader spectrum compared with the full-length PlyCD. Endolysin–vancomycin synergy was observed. *Ex vivo assays:* Recombinant endolysin was able to kill vegetative cells in the colon of mice with approximately 2 log reduction after 1 h of incubation.	(32)
CD11 and CDG endolysins; *In vitro* assays	Both endolysins were highly active against *C. difficile*.	(33)
Recombinant protein composed of the phiCD2 endolysin catalytic domain (EAD) and human alpha-defensin functional domain (HD_5_); *in vitro* assays; Mouse model of CDI	*In vitro assays:* Recombinant endolysin killed several *C. difficile* ribotypes, reduced TcdB cytotoxicity, and had lower MICs (0.78 μg/ml) than metronidazole and vancomycin. *In vivo:* Treatment reduced CDI symptoms and reduced *C. difficile* and fecal toxin load in mice. Treated mice recovered while 40% of untreated mice died.	(34)
phiMMP01 cell wall hydrolase; *in vitro* assays	Removing the CBD and keeping only the EAD increased lytic activity and expand the activity spectrum. Inhibition of spore outgrowth. Active at various pH and temperatures.	(35)
Endolysin CD16/50L; *in vitro* assays	Removing the CBD increased activity and expanded the host spectrum, CBD remains trapped with cellular debris. Endolysin can have off-target hydrolyzation on other clostridial relatives.	(36)
Endolysin Ecd09610; and its domain variants; *in vitro* assays	The two C-terminal domains hold the lytic activity and showed the best clearing of the culture. The domain variants were thermoresistant up to 100°C and can be easily produced at high concentrations (contrarily to full-size lysin). Stable after lyophilization. Weak lytic activity was found in some related bacteria.	(37)
*Lys6356* and its EAD; *in vitro* assays	Endolysins can be used after spore treatment with germinants and inhibits spore outgrowth. The use of taurocholic acid and glycine did not affect Lys6356 activity. Calcium which is present in the gut and is massively released during spore germination inhibited endolysin activity *in vitro*.	(38)

## Phage cocktails are more efficient than single phage treatments

The lytic potential of different *C. difficile* phages has been assessed in several *in vitro* assays. The main findings are that phages kill vegetative cells efficiently, reducing bacterial counts by several logs (19, 21, 23, 25). However, one common observation with single phage treatments has been culture rebound due to lysogeny. This was well described with phage phiCD27 used in batch fermentations and *in vitro* gut models (19, 20). A well-known method to limit the rise of phage resistance is to use phage cocktails (9). The administration of single or multiple phage combinations was compared *in vitro* (21, 23). Cocktails comprising 3–4 different phages better prevented the culture regrowth compared with cocktails comprising a single phage. Interestingly, some cocktails were shown to prevent *C. difficile* biofilm formation (22). Biofilms are complex ecosystems generally comprising multiple bacterial species embedded into a matrix composed of extracellular polymeric substances (EPS), such as polysaccharides, DNA, amyloids, lipids, and proteins (39). Some phages possess depolymerase activity at the tip of their tail that can degrade EPS and biofilms (40). Although *C. difficile* phages with depolymerase activity have never been described, some phages were shown to penetrate and destabilize already-formed biofilms. However, complete eradiction of *C. difficile* from an already established biofilm was not observed (22). It is worth mentioning that the results varied depending on the targeted *C. difficile* strain, suggesting that optimization of the cocktails would be necessary on a strain-specific basis. The behavior of phages in the presence of human colonic cells in culture was also investigated, and a higher lytic activity of phage phiCDHS1 was observed in the presence of cultured HT-29 cells. This was explained by the high-phage adsorption to the cell line on which *C. difficile* also adheres, promoting phage–bacteria interactions (24).

## Temperate phages are generally unable to completely cure CDI in animal models

The hamster model of CDI has been used to assess the efficacy of temperate phages. The first study was reported by Ramesh et al. (18). The authors found that simultaneous administration of ϕCD140 along with *C. difficile* spores greatly increased the survival of infected hamsters, as opposed to untreated animals that died within 72 h (18). While this first study was globally successful, one of the treated hamsters died due to the rise of phage resistance, which had been attributed to phage receptor mutation or lysogeny. *In vivo* lysogeny was later confirmed in hamsters during treatment with phage phiCD119. The proportion of lysogenic *C. difficile* clones was shown to increase over time, and by day 4 of the experiment, only lysogens could be isolated (41). These results revealed the incapacity of single-temperate phages to treat CDI in hamsters. To circumvent the problem of lysogeny, two- to four-phage cocktails were tested (22). While the luminal bacterial loads were reduced by at least 4 logs, the four-phage cocktail, administered every 8 h, prolonged hamster survival by 3 days compared with untreated animals. Ultimately, all animals died of CDI. Due to hamsters being highly sensitive to *C. difficile* and its toxins, the relevance of this model to the human condition has been questioned (42). Therefore, alternative CDI models, such as the wax moth larvae *Galleria mellonella*, have been recently developed. An optimized four-phage cocktail was tested against infection with a ribotype 014/020 *C. difficile* strain in *G. mellonella* larvae. Prophylactic single-dose cocktail administration (10^6^ PFU) prior to bacterial inoculation with 10^5^ CFU of vegetative cells led to complete protection and survival of all insects 60 h post-infection, though ~2-log bacterial counts were still detected at the end of the experiment. When phages were administered simultaneously with bacteria, survival dropped to 72%, whereas treatment with phages 2 h post-infection led to 30% survival after 60 h, and all larvae died at the end of the experiment. Multiple phage doses as well as vancomycin prophylaxis before infection and prior to phage treatment improved the outcome. These results show that the timing of phage inoculation is crucial for the efficacy of phage therapy, and that prophylactic regimens are more effective than remedial regimens, as observed *in vitro* (22, 26). The *G. mellonella* model is easier to manipulate than hamsters or mice and can be useful to test different hypotheses. Whether the observations made with this model are readily transferable to a more complex ecosystem like the mammalian gut remains to be demonstrated.

## Genetically engineered phages

The first case of a genetically engineered *C. difficile* phage involved the deletion of the genes coding for the *cI* repressor and the integrase in phage phiCD24-2 (43), therefore creating the first strictly lytic *C. difficile* phage (25). The authors also produced a crPhage carrying a mini-CRISPR array targeting the toxin locus, as well as a phage combining both modifications. The crPhages had significantly increased killing power and showed reduced lysogeny while the strictly lytic phages did not lead to lysogeny *in vitro*. The strictly lytic phage was not better than the wild-type phage at reducing bacterial counts *in vitro*, contrary to the *in vivo* condition where it performed better. Unexpectedly, regrowth of *C. difficile* was observed in the mouse gut with the recombinant phages. Clones recovered from fecal samples were lysogens of the modified phages. This unexpected result could not be clearly explained, but it was hypothesized that endogenous prophages could potentially have complemented the lost functions in the recombinant phages (25).

## Phage tail-like particles are potent antimicrobials

**Figure 1:**
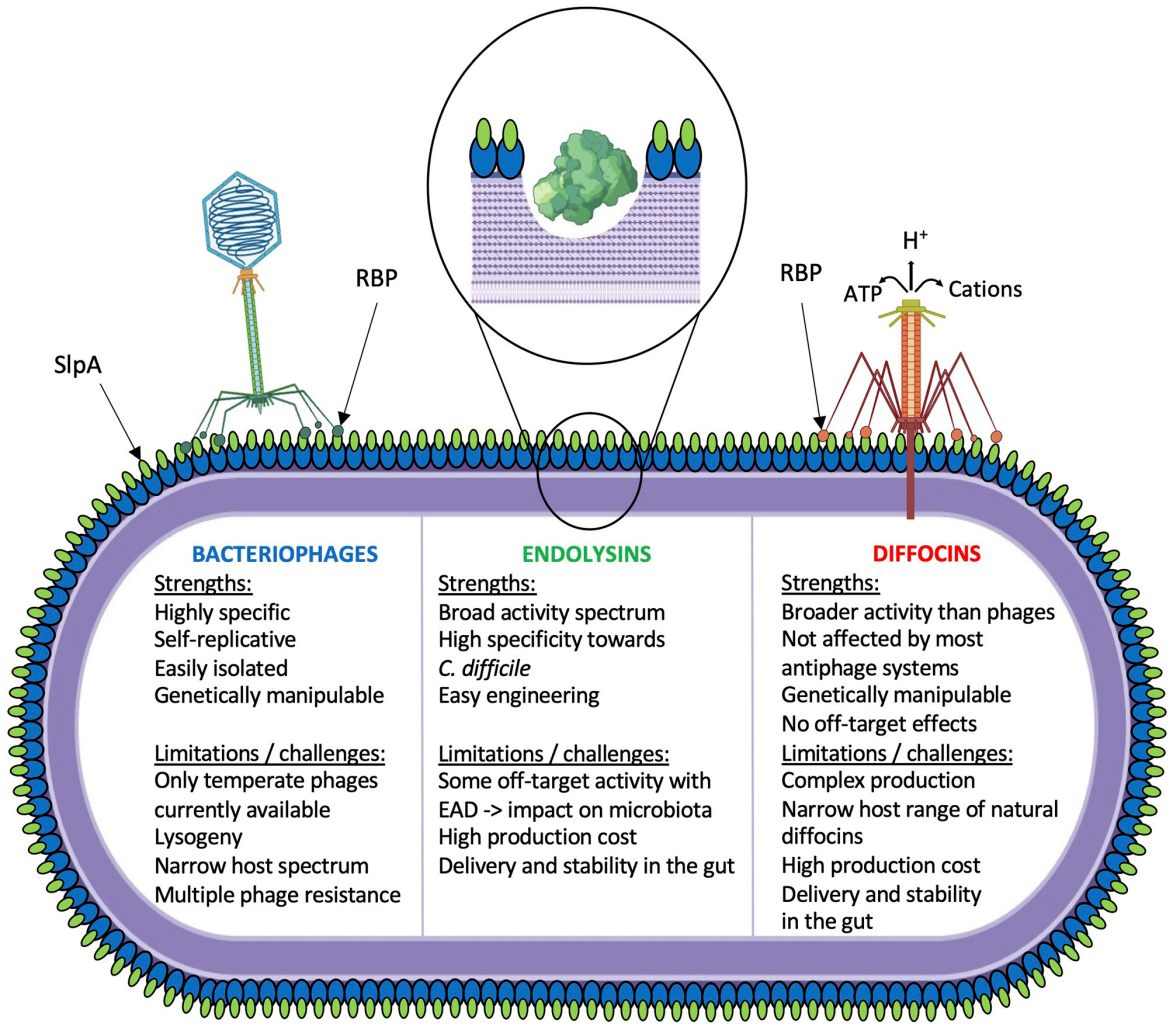
Schematic overview of current phage-based investigations. Strengths and limitations/challenges for each strategy are indicated.

Phage tail-like particles are high molecular weight bacteriocins produced by several bacteria as a mechanism to compete with closely related species. Several strains of *C. difficile* produce these particles, which have been called diffocins (29, 44–47). Diffocins are genetically and structurally related to contractile phage tails but without a capsid. Hence, they do not carry genetic material and cannot replicate. Once adsorbed to a susceptible host via a specific receptor, contraction of the tail sheath leads to perforation of the cell membrane by the inner tail tube, causing leakage of the cell content and death (Figure 1) (48). Heterologous expression of functional recombinant diffocins has been successful in *Bacillus subtilis* (27). However, akin to their phage homologs, the host range of diffocins is generally narrow. To circumvent this, a genetically engineered diffocin was created by replacing its receptor-binding protein (RBP) with another from phi027, a prophage conserved in the genome of the R20291 and other R027 epidemic strains (28). This hybrid diffocin, called Avidocin-CD291 (or Av-CD291), was redirected toward strain R20291 and was able to kill all ribotype 027 strains tested *in vitro*, in addition to one or more isolates of ribotype 001, 015, and 087 strains (28). A turning point was reached when diffocins were shown to recognize their bacterial host via binding to the surface layer protein A (SlpA) (49). The authors demonstrated that diffocins' RBPs specifically bind to certain SlpA isoforms (or SLCTs, for surface layer cassette types). Interestingly, they also showed the interchangeability of the diffocin RBPs, and genetically engineered avidocins could be redirected toward different *C. difficile* strains based on their SLCT status (49). Most importantly, the efficacy of Av-CD291 was tested in a mouse model of *C. difficile* spore transmission that mimics the natural human-to-human transmission. Prophylactic administration of purified Av-CD291 in the drinking water completely prevented the colonization of mice, and no *C. difficile* could be detected in fecal samples, as opposed to the placebo group (28). There was no significant change in the composition of the microbiota, suggesting that avidocins are very specific to *C. difficile* and do not disturb the microbiota (28, 29). This study also revealed that Av-CD291 administration in mice did not disturb colonization resistance to *C. difficile* or vancomycin-resistant *Enterococcus faecium* (28).

## Cell wall-degrading enzymes as antimicrobials

Phage endolysins are also promising alternatives to whole phages (Figure 1). Tailed phages must break up the cell wall to escape their host at the end of the lytic cycle. The canonical holin–endolysin pathway involves a small protein, the holin, that accumulates into the cytoplasmic membrane until a programmed time of the lytic cycle at which point it forms pores into the cell membrane. This allows the endolysin to escape the cytoplasm and reach the peptidoglycan layer that it hydrolyzes from within until lysis (50). Gram-positive phage endolysins are generally composed of an enzymatically active domain (EAD) and a cell wall-binding domain (CBD). Most *C. difficile* phage endolysins are N-acetylmuramoyl-L-alanine amidases (35, 51). When purified endolysin is added extracellularly to a bacterial suspension *in vitro*, rapid lysis occurs in a matter of 10–20 min. A few *C. difficile* phage endolysins have been cloned and characterized (Table 1), CD27L being the first to be described (30). Of note, one common observation that has been reported with all *C. difficile* phage endolysins is that the catalytic domain of the enzyme alone (EAD) is sufficient for full activity. In fact, the removal of the CBD was reported to increase the lytic activity of *C. difficile* (30–32, 36, 38). It was also shown that the CBD from the CD16/50L endolysin was responsible for trapping the endolysin within cellular debris after lysis. It was hypothesized that the CBD domain prevents the endolysin from being released freely into the environment upon cell lysis, therefore preserving uninfected bacteria that can serve as hosts for subsequent phage infection (36). Full-length endolysins were shown to be very specific toward *C. difficile*, as little or no activity was noted on other commensal bacteria, including related Clostridia. However, most truncated endolysins comprising only the EAD displayed a slightly broader host range, killing all *C. difficile* isolates tested, in addition to a few other species, in particular *Clostridium sordellii, Clostridium bifermentans, Bacillus subtilis*, and *Listeria monocytogenes* (30–32, 36, 38).

An interesting feature of gram-positive endolysins is their modular architecture that allows interchanging EAD and CBD. The LHD is a lysin–human defensin fusion protein that results from the fusion of the CBD from phage phiC2 endolysin and the functional domain from the human α-defensin 5 (HD_5_) (34). LHD was very active on several *C. difficile* strains of different ribotypes, and the minimum inhibitory concentration (0.78 μg/ml) was >4 times lower than that of metronidazole and vancomycin. Interestingly, LHD inhibited the glycosylation activity and toxicity of TcdB, as shown with HD_5_ (52). Furthermore, treatment of mice infected with the R20291 epidemic strain twice a day for 7 consecutive days with LHD rescued all treated mice from death, as opposed to 60% survival for control mice. Toxin levels and the number of spores were also reduced in the treated group. Interestingly, pre-treatment of bacteria with vancomycin increased the lytic activity of the endolysin PlyCD_1 − 174_, suggesting a synergistic effect as described with phages (53). In addition, PlyCD_1 − 174_ endolysin was shown to be active *ex vivo* in a complex mouse fecal environment (32).

## Discussion

Aside from the common challenges that phage therapy faces in general, such as safety, efficacy, the lack of data from clinical trials, resistance, regulatory hurdles, and patentability, several limitations are particularly relevant to CDI. The most urgent problems to address are as follows: (i) the lack of strictly lytic phages, (ii) the narrow host range of current phages, and (iii) the problem of phage resistance. Alternatives to whole phages also have their limitations, such as (vi) the difficulty to produce avidocins or endolysins on a large scale, (vii) the stability of avidocins and endolysins in the intestinal environment, and (viii) the off-target activity of genetically engineered endolysins. However, there are potential solutions to these limitations.

The lack of strictly lytic phages is the most critical problem with *C. difficile* phages, and further screening of environmental samples is not the solution. The most reasonable strategy is the genetic engineering of temperate phages. Such phages have been successfully created and tested in other phage–host models (54, 55), and a human patient infected by *Mycobacterium abscessus* has been treated with one of them (16). The first report of a genetically engineered *C. difficile* phage has proven the feasibility of this approach, although further research is required to better characterize the behavior of genetically engineered phages *in vivo*. It will be particularly important to investigate how therapeutic phages interact with the highly prevalent and diverse endogenous prophages in *C. difficile* genomes (56).

Another important limitation of current *C. difficile* phages is their narrow host range, implying that multiple phages will need to be combined into cocktails to cover the most clinically relevant strains of *C. difficile* (21). Due to recent advances in our understanding of host recognition by *C. difficile* phages, the surface layer protein A (SlpA) seems to be a general receptor used by many phages and diffocins (28, 49, 57–59). It is, therefore, reasonable to foresee the selection of phages based on their RBP to target *C. difficile* strains expressing specific SlpA isoforms. Although RBP can be identified in phage genomes using bioinformatics tools, it will be important to determine if additional phage proteins participate in host recognition or if other receptors exist, as suggested in one study (58).

Resistance is always a critical concern when undertaking phage therapy (12, 60). Several mechanisms of phage resistance exist (61), and an important one is the mutation of the phage receptor. Work on diffocins led to the isolation of two spontaneous *C. difficile* mutants that had an SNP causing severe truncation of the SlpA protein, leading to full resistance to diffocins (49). If strictly lytic phages are developed for CDI treatment, phage resistance through mutation of SlpA might occur as well. However, *in vitro* and *in vivo* data showed that loss of slpA comes with a huge fitness cost. Indeed, *slpA* mutants produce less toxins, sporulate less, are more sensitive to antimicrobial peptides, and are avirulent (49, 62). Therefore, phage resistance through receptor mutation would be compensated by loss of virulence, facilitating patient recovery.

Additional factors that can affect the success of a phage infection will also need to be considered. For instance, active antiphage mechanisms have been described in *C. difficile*, including restriction-modification systems (63) and a type I CRISPR-Cas system (64). A superinfection exclusion mechanism mediated by the phase-variable cell wall protein CwpV has also been described, although its impact on phage resistance *in vivo* needs to be investigated (65). The recent identification of functional anti-CRISPR systems in several *C. difficile* phage genomes (66) suggests that CRISPR-mediated interference could potentially be short-circuited by selecting anti-CRISPR-containing phages or by incorporating anti-CRISPR genes into genetically modified phages. Importantly, incorporation of CRISPR cassettes into cargo phage genomes to target the host cell [e.g., toxin or other virulence genes (25)] will require considering the fact that some naturally occurring lysogens might express anti-CRISPR systems and therefore negatively interfere with the engineered phages.

Avidocins are very appealing alternatives to whole phages, but their large-scale production will be challenging because they are a complex assemblage of multiple components. On the other hand, endolysins are much simpler to produce and have a broader host range, which is a clear advantage. However, off-target killing might be a problem, as some of the other clostridial species that can be lysed by certain recombinant endolysins are beneficial species, such as *C. scindens* which has been shown to protect against CDI through primary bile acid conversion (67).

Sporulation of *C. difficile* is also one important hurdle in the treatment of CDI. Spores are naturally produced in the gut during infection and become resistant to most antimicrobials. Hence, combination therapy that includes spore germinants could promote germination, therefore ensuring maximum killing of the infected strain. This strategy has, however, been shown to interfere with endolysin activity, as the massive release of calcium during germination is shown to inhibit the activity of LysCD6356, at least *in vitro* (38). The importance of this observation to the *in vivo* condition requires further investigation. Nevertheless, even if residual spores remain after phage-based treatment, the risk of relapse should be lower than with conventional antibiotics because the gut microbiota will be spared in the process.

In conclusion, we are still a few steps from a viable phage-based product to fight CDI, but recent progress in our understanding of phage–host interactions and the development of more efficient molecular tools to genetically engineer phages will certainly lead to exciting advances in the next few years.

## Author contributions

AU: Conceptualization, Writing—original draft, Writing—review and editing. L-CF: Conceptualization, Funding acquisition, Project administration, Resources, Supervision, Writing—original draft, Writing—review and editing.
